# Chicken fillets subjected to UV‐C and pulsed UV light: Reduction of pathogenic and spoilage bacteria, and changes in sensory quality

**DOI:** 10.1111/jfs.12421

**Published:** 2017-11-23

**Authors:** Anette McLeod, Kristian Hovde Liland, John‐Erik Haugen, Oddvin Sørheim, Kristine S. Myhrer, Askild L. Holck

**Affiliations:** ^1^ Nofima, Norwegian Institute of Food, Fisheries and Aquaculture Research Ås Norway

## Abstract

We have compared the efficacy of continuous ultraviolet (UV‐C) (254 nm) and pulsed UV light in reducing the viability of *Salmonella* Enteritidis, *Listeria monocytogenes*, *Staphylococcus aureus*, enterohemorrhagic *Escherichia coli*, *Pseudomonas* spp., *Brochothrix thermospacta, Carnobacterium divergens*, and extended‐spectrum β‐lactamase producing *E. coli* inoculated on chicken fillet surface. Fluences from 0.05 to 3.0 J/cm^2^ (10 mW/cm^2^, from 5 to 300 s) used for UV‐C light resulted in average reductions from 1.1 to 2.8 log cfu/cm^2^. For pulsed UV light, fluences from 1.25 to 18.0 J/cm^2^ gave average reductions from 0.9 to 3.0 log cfu/cm^2^. A small change in the odor characterized as sunburnt and increased concentration of volatile compounds associated with burnt odor posed restrictions on the upper limit of UV treatment, however no sensory changes were observed after cooking the meat. Treatments under modified atmosphere conditions using a UV permeable top film gave similar or slightly lower bacterial reductions.

**Practical applications:**

Ultraviolet (UV) light may be used for decontaminating the surface of food products and reduce viability of pathogenic and spoilage bacteria. Exposure of raw chicken fillet surface to various doses of continuous UV‐C or pulsed UV light proposed in the present work represent alternatives for microbiological improvement of this product. Chicken fillets can be treated in intact packages covered with UV permeable top film, thus avoiding recontamination of the meat. UV‐C light treatment is a low cost strategy with low maintenance, whereas pulsed UV light involves more elaborate equipment, but treatment times are short and less space is required. Both methods can be helpful for producers to manage the safety and quality of chicken fillets.

## 1 INTRODUCTION

The desired long shelf life in today's food industry has led to increasing demands in the development of methods for improving microbial safety and quality. According to the Food and Agriculture Organization of the United Nations (FAO), the average annual consumption of chicken meat pro capita worldwide increased from 10.2 kg in 1999 to 13.8 kg in 2015 (FAO, 2015). The global meat consumption is projected to rise more than 4% per person over the next 10 years, and for poultry it is predicted to rise more than 10% (Organisation for Economic Co‐operation and Development/Food and Agriculture Organization of the United Nations, 2016). As live poultry animals contain microorganisms on their skin, feathers, and in their digestive tract, contamination of the carcasses during slaughtering procedures cannot be completely avoided when live animals are converted to meat for consumption.

Food contamination is a major global burden because of foodborne illnesses that can result from it. Poultry may be the vector of *Salmonella* spp., *Campylobacter* spp., *Staphylococcus aureus*, *Listeria monocytogenes*, Shiga toxin‐producing *Escherichia coli*, and other pathogens (Capita, Alonso‐Calleja, Garcia‐Fernandez, & Moreno, 2002; Hafez, 1999; Zhao et al., 2001). The first two mentioned are the most common causes of human foodborne bacterial diseases linked to poultry (European Food Safety Authority [EFSA], 2015; Hafez, 2005). According to the Community Summary Reports of the EFSA and the European Centre for Disease Prevention and Control, 2008, campylobacteriosis and salmonellosis accounted for 214,779 and 82,694, respectively, confirmed human cases in the EU (EFSA, 2015). The number of confirmed listeriosis cases in humans was 1,763, where a high fatality rate of 15.6% was reported among the cases. Antibiotic‐resistant bacteria, such as the extended‐spectrum beta‐lactamase (ESBL)‐producing *E. coli*, have become a growing public health threat (Briongos‐Figuero et al., 2012; Lu et al., 2012; Picozzi et al., 2013; Pitout, 2010). The ESBL‐producing strains are feared as they produce the enzyme beta‐lactamase that has the ability to break down commonly used antibiotics like penicillins and cephalosporins, and render them ineffective for treatment. In 2014, the World Health Organization (WHO) warned that the antibiotic resistance crisis is becoming dire, with diseases that have been curable for decades becoming increasingly difficult to treat (Michael, Dominey‐Howes, & Labbate, 2014; WHO, 2014). The presence of ESBL genes has been clearly documented in Enterobacteriaceae isolated from food‐production animals, and especially from chickens (Machado, Coque, Canton, Sousa, & Peixe, 2008; Overdevest et al., 2011; Smet et al., 2008). Occurrence of cephalosporin‐resistant *E. coli* on poultry in Norway ranged from 8 to 43% (Mo et al., 2014).

Food rendered unfit for human consumption because of product spoilage results in significant economic losses when products must be removed from the market. The accumulation of metabolic by‐products or the action of extracellular enzymes produced by spoilage bacteria multiplying on these foods, leads to deterioration like discoloration, texture change, and formation of off‐flavors, off‐odors, and slime. The meat acquires an offensive odor when the bacterial flora reaches about 10^7^ cfu/cm^2^ of the surface, and when reaching 10^8^ cfu/cm^2^, the surface becomes slimy (Borch, Kant‐Muermans, & Blixt, 1996; Holck, Pettersen, Moen, & Sorheim, 2014; Molin, 2000). The natural microflora on chicken fillets has been identified (Holck et al., 2014), and common spoilage microorganisms when stored aerobically at 4°C are *Pseudomonas* spp., *Brochothrix* spp., and Enterobacteriaceae. A widely used strategy for increasing shelf life of poultry meat is modified atmosphere packaging (MAP) (Holck et al., 2014; van Velzen & Linnemann, 2008). Storage with high CO_2_ (70% CO_2_, 30% N_2_) can lead to lactic acid bacteria like carnobacteria dominating the flora (Holck et al., 2014; Vihavainen et al., 2007). Although some strains of carnobacteria show little influence on the sensory properties of a product, others can spoil the product (Laursen et al., 2005; Leisner, Laursen, Prevost, Drider, & Dalgaard, 2007).

Various physical and chemical methods to reduce microbes on poultry products have been studied, such as water spraying, air chilling, ultrasound, irradiation, trisodium phosphate, and lactic acid (Capita et al., 2002; Loretz, Stephan, & Zweifel, 2010). Potential disadvantages using these methods are sensory changes, deterioration of product appearance and quality, and safety concerns. In recent years, there has been a growing interest in using ultraviolet (UV) light for decontamination of poultry. UV light is widely known for its germicidal effect by damaging nucleic acids (Kowalkski, 2009). The high energy associated with short‐wavelength UV energy (UV‐C), primarily at 254 nm, is absorbed by cellular RNA and DNA. This energy absorption initiates a reaction between adjacent pyrimidine bases to form dimer lesions, which in turn inhibit replication and transcription in cells (Harm, 1980; Weber, 2005).

As a means for controlling surface microorganisms on food products, regulations in conjugation with using conventional continuous UV‐C light (henceforth referred to as UV‐C light) in the United States are given by the U.S. Food and Drug Administration (FDA) (FDA, 2010). UV‐C light can be employed in Europe, however, in Germany the use is limited to water, fruit and vegetable products, and stored hard cheeses (Anonymous, 2000). Decontamination of raw boneless, skinless chicken, or broiler breast fillets by the use of UV‐C light has been reported to reduce bacterial counts of various pathogens by 0.6 to 1.7 log depending on the conditions used (Chun, Kim, Lee, Yu, & Song, 2010; Haughton et al., 2011b; Isohanni & Lyhs, 2009; Sommers, Scullen, & Sheen, 2016). High intensity pulsed UV light has been approved by the FDA up to 12 J/cm^2^ (FDA, 2010). The UV energy spectrum of pulsed UV light consists of a continual broadband spectrum from deep UV to infrared light, especially rich in UV range below 400 nm, which is germicidal. In addition to creating dimer lesions, pulsed UV light has been proposed to cause cell damage and cell death by inducing damage of the cell membrane and to cause rupture of the bacteria by thermal stress (Krishnamurthy, Tewari, Irudayaraj, & Demirci, 2010; Takeshita et al., 2003; Wekhof, 2000). The use of this technology for food decontamination has previously been reviewed (Demirci & Panico, 2008; Gomez‐Lopez, Ragaert, Debevere, & Devlieghere, 2007). Pathogen reduction on boneless skinless chicken breast has been reported to vary from 1.2 to 2.4 log depending on the conditions used (Keklik, Demirci, & Puri, 2010; Paskeviciute, Buchovec, & Luksiene, 2011). Several investigations have demonstrated the effectiveness of UV light on microbial reduction *in vitro*, and a wide range of bacterial species were reduced by 5–7 log when treated on petri dishes under different conditions (Farrell, Garvey, Cormican, Laffey, & Rowan, 2010; Gomez‐Lopez, Devlieghere, Bonduelle, & Debevere, 2005; Paskeviciute et al., 2011; Rowan et al., 1999).

The objective of our investigation was to study and compare the efficacy of UV‐C and pulsed UV light against pathogens and bacteria often found as natural contaminants on fresh chicken meat, of which several of the species have not previously been investigated for UV light treatment on food. To our knowledge, studies on UV light exposure of intact packages of MAP‐chicken fillet for bacterial reduction have not been reported, thus we aimed at undertaking this issue using a UV permeable top film. We also aimed at determining whether the UV light treatments had adverse effects on the sensory quality of chicken fillets.

## 2 MATERIALS AND METHODS

### 2.1 Bacterial strains, media, and growth conditions

The bacterial strains used in this work are listed in Table 1. The strains were maintained at −80°C in their respective media supplemented with 20% glycerol (vol/vol). Rifampicin resistant (Rif^R^) derivatives were prepared for all isolates by growing strains in liquid media containing 200 µg/ml rifampicin as described by Heir et al. (2010), except for the ESBL‐producing *E. coli* strains already resistant to several types of antibiotics. Growth experiments using a Bioscreen C instrument (Labsystems) where the Optical Density (OD) at 600 nm was monitored, showed no significant difference in growth between the original strains and their Rif^R^ mutants in their respective media and growth conditions. The different bacterial strains of each species were cultured separately. *Carnobacterium divergens* was grown in cystein‐deMan Rogosa Sharpe broth (cMRS, Oxoid) with 200 µg/ml rifampicin (Sigma‐Aldrich; 48 hr incubation, 30°C), ESBL‐producing *E. coli* in Brain Heart Infusion broth (BHI; Oxoid) with 50 µg/ml ampicillin (Sigma‐Aldrich; 16 hr incubation, 37°C), and tryptic soy broth (TSB, Oxoid) with 200 µg/ml rifampicin was used for *Pseudomonas* spp. (16 hr incubation, 30°C), *Brochothrix thermospacta* (48 hr incubation, 30°C), *Salmonella* Enteritidis, *L. monocytogenes*, *S. aureus*, and EHEC (16 hr incubation, 37°C). Before decontamination experiments, bacterial cultures of each of the different strains of the same species were mixed in equal amounts, for example, bacterial cultures of each of the four strains of *L. monocytogenes* were mixed 1:1:1:1. An exception was *E. coli*, for which the ESBL‐producing *E. coli* strains and the EHEC strains were separated from each other.

**Table 1:** Strains used in this study

Bacterial species	Strain name^a^	Reference/source/strain/other
*Pseudomonas* spp.	MF6041	Chicken fillet
	MF6042	Chicken fillet
	MF6043	Chicken fillet
	MF6044	Chicken fillet
*B. thermospacta*	MF6045	Chicken
	MF6047	Chicken
	MF6049	ATCC11509^b^
*C. divergens*	MF3036	DSM20623^c^
	MF6031	Chicken fillet
	MF6032	Chicken fillet
	MF6034	Chicken fillet
	MF6038	Chicken fillet
ESBL‐producing *E. coli*	MF5658	Chicken^d^
	MF5660	Chicken^d^
	MF5664	Chicken^d^
	MF5670	Broiler^d^
	MF5674	Broiler^d^
*S*. Enteritidis	MF3817	1049‐1‐99^d^
	MF3818	Poultry, 61–358‐1^e^
	MF3824	ATCC13076^b^
*L. monocytogenes*	MF3508	2230/92 (Nesbakken, 1995)
	MF3509	167 (Blom et al., 1997)
	MF3510	187 (Blom et al., 1997)
	MF3571	EGD‐e (Glaser et al., 2001)
*S. aureus*	MF2123	ATCC25923^b^
	MF2124	ATCC12600^b^
	MF2125	ATCC6538^b^
Enterohemorrhagic *E. coli* (EHEC)	MF3572	O103, fermented sausage, linked to outbreak in Norway 2006 (Schimmer et al., 2008)^f^
	MF3574	ATCC43895^b^, O157:H7
	MF3576	O111:H^‐^, semi‐dry fermented sausage, outbreak Australia 1995 (Paton et al., 1996)^g^
	MF5554	O145 (McLeod et al., 2016)

^a^Antibiotic resistant strains. All strains were grown in their respective medium with 200 µg/ml rifampicin, except ESBL‐producing *E. coli* grown in medium with 50 µg/ml ampicillin.

^b^ATCC, American Type Culture Collection, Manassas, VA, USA.

^c^DSM, Deutsche Sammlung von Microorganismen und Zellkulturen, Braunschweig, Germany.

^d^Kindly received from the Norwegian Veterinary Institute, Oslo, Norway.

^e^Kindly received from the Technical University of Denmark, the National Veterinary Institute, Denmark.

^f^Kindly received from the Norwegian School of Veterinary Science, Oslo, Norway.

^g^Kindly received from Statens Serum Institut, Copenhagen, Denmark.

### 2.2 UV illumination experiments of chicken and agar surface inoculated with bacterial cells

Fresh skinless chicken breast fillets were purchased from local Norwegian supermarkets. The meat was cut into pieces of 10 cm^2^, and one side of the chicken was inoculated by spreading 15 µL suspension of a multi strain mix of one species (described above) to obtain bacterial levels of 10^5^–10^7^ cfu/cm^2^. The inoculated chicken samples were left at room temperature to dry for 1 hr prior to UV light treatment. To assess the indigenous background flora of the chicken, uninoculated samples were also analyzed. For *in vitro* illumination experiments, serial 10‐fold dilutions of each multi strain mix were made and plated onto the respective agar media (described below). In the UV‐C light experiments, samples were treated in a custom made aluminum chamber (1.0 × 0.5 × 0.6) m^3^ equipped with two UV‐C lamps (UV‐C Kompaktleuchte, 2x95 W, BÄRO GmbH, Leichlingen, Germany) in the ceiling. The UV‐C light was emitted essentially at 253.7 nm, measured using a UVX Radiometer (Ultra‐Violet Products, Ltd., Cambridge, UK) equipped with a UV‐C sensor (model UVX‐25, Ultra‐Violet Products). Both sample distance (6 cm) from the lamps and duration of the exposures were chosen with aim to be relevant for industrial production lines. Exposures were thus at 10 mW/cm^2^, which is close to a maximum when using commercial lamps, for 5, 10, 30, 60, or 300 s, giving fluences of 0.05, 0.1, 0.3, 0.6, 3.0 J/cm^2^, respectively. For the pulsed UV light experiments, a semiautomated intense pulsed UV system instrument XeMaticA‐SA1L (SteriBeam Systems GmbH, Kehl‐Kork am Rhein, Germany) was used. Samples were placed in the instrument chamber at a 6.5 cm distance from the xenon lamp (19 cm), which was water cooled, had an aluminum reflector (10 cm × 20 cm), and the spectral distribution was 200–1,100 nm, with up to 45% of the energy being in the UV‐region (maximal emission at 260 nm). The fluences were set according to the manufacturers specifications, and were adjusted to 1.25 J/cm^2^ (low) or 3.6 J/cm^2^ (high). The lowest level of exposure would result in limited bacterial reductions, and fluences up to and above the limit value of 12 J/cm^2^, which is the maximum permitted dose by FDA (FDA, 2010), were tested. Samples were exposed either once to the low pulse, or one, three, or five times to the high pulse (3.6, 10.8, or 18.0 J/cm^2^, respectively). Three parallels of both treated samples and untreated controls were produced for each experiment, and the experiments were repeated three times on different days.

For ESBL‐producing *E. coli* and *C. divergens*, UV light treatments were also performed under modified atmosphere conditions as follows: Chicken sample with inoculated bacteria placed in a tray was packaged using a Polimoon 511VG tray sealing machine (RPC Promens AS, Kristiansand, Norway) and UV permeable top film with 65 µm thickness and an ethylene vinyl alcohol (EVOH) barrier layer (Opalen 65, Bemis, Oshkosh, WI). A gas mixture of 60% CO_2_ and 40% N_2_ (AGA, Oslo, Norway) was used for the packages. The film had an oxygen transmission rate (OTR) of 5 ml/m^2^/24 hr/atm at 23°C/50% RH, and the trays of dimension 208 × 146 × 32 mm had a barrier layer of high density polyethylene (HDPE; RPC Promens 528) with an OTR of 3.5 ml/m^2^/24 hr/atm at 23°C/50% RH. Intact packages (MAP‐chicken) were exposed to UV light doses similar to the chicken samples treated in air (unpackaged chicken), allowing for comparison of bacterial reduction between the two. Three parallels of both treated samples and untreated controls were produced for each experiment. The experiments were repeated three times on different days.

Temperatures were measured using a Raynger MX infrared thermometer (Raytek Corporation, Santa Cruz, CA). Samples were subjected to microbial and physiochemical analyses as described below. The experiments with pathogens were performed in a Biosafety level 3 pilot plant.

### 2.3 Microbial analyses

Chicken samples were added 90 ml of peptone water and the samples were homogenized for 1 min in a stomacher (AES Smasher, AES Chemunex, Bruz, France). Serial 10‐fold dilutions from each sample were prepared. Quantification of *C. divergens* (cfu/cm^2^) was performed using a Whitley Automatic Spiral Plater (Don Whitley Scientific, Ltd., West Yorkshire, UK) on cMRS agar (Oxoid) with 200 µg/ml rifampicin (48 hr incubation, 30°C), ESBL‐producing *E. coli* on BHI (Oxoid) with 50 µg/ml ampicillin (16 hr incubation, 37°C), and tryptic soy agar (TSA, Oxoid) with 200 µg/ml rifampicin was used for *Pseudomonas* spp. (16 hr incubation, 30°C), *B. thermospacta* (48 hr incubation, 30°C), *S*. Enteritidis, *L. monocytogenes*, *S. aureus*, and EHEC (16 hr incubation, 37°C). The number of colonies were determined using an automatic plate reader, and the detection limit was 20 cfu/cm^2^. Since rifampicin resistant strains were used, the indigenous background flora on the chicken was negligible.

### 2.4 Packaging film analyses

The UV permeable top film Opalen 65 was evaluated for its ability to transmit UV light by measuring UV light at 254 nm (described above). The extended O_2_ barrier properties of the top film was evaluated by using empty packages with 100% N_2_ that were initially exposed to four different UV‐C and pulsed UV light treatments up to 10.8 J/cm^2^ in addition to an untreated control, with five packages per treatment. The packages were analyzed for concentrations of residual oxygen at packaging and after 21 days of storage with a Dansensor Checkmate 3 (Dansensor, Ringsted, Denmark). The top films of the trays used for oxygen analysis were also evaluated for structural damages by UV light by scanning electron microscopy, where the samples were mounted on an aluminum stub using double‐sided tape coated with carbon, before being coated with gold/palladium using a SC7640 auto/manual high resolution sputter coater (Quorum Technologies, Ashford, UK). An EVO‐50‐EP environmental scanning electron microscope (Zeiss, Cambridge, UK) was used to study the samples at a magnification of 8000×.

### 2.5 Preparation of chicken samples for sensory analyses

Refrigerated fresh skinless chicken breast fillets obtained from a local producer were mixed to achieve an equal number of cfu per cm^2^ on the surface. One set of chicken samples were exposed to UV light in air (unpackaged chicken), and were thereafter packaged in modified atmosphere, while a parallel set of chicken samples were exposed to UV light under modified atmosphere (MAP‐chicken), as described above. None of these chicken samples were inoculated with bacterial culture, and both sample sets were then stored at 4°C for 6 days before being used for the sensory analyses described below. The color stability of the chicken fillets were evaluated by visual inspection of the chicken before and after UV light exposure, and after storage.

### 2.6 Sensory evaluations

Descriptive sensory profiling was conducted by a trained sensory panel of 10 assessors at Nofima AS, Norway, according to Generic Descriptive Analysis (Lawless & Heymann, 2010). All panelists were selected and trained in accordance with ISO 8586:2012 (International Organisation for Standardisation, 2007). The following chicken samples treated in air and under modified atmosphere were prepared: untreated control, chicken exposed to UV‐C at fluence 0.1 J/cm^2^ (10 s at 10 mW/cm^2^), chicken exposed to UV‐C at fluence 0.6 J/cm^2^ (60 s at 10 mW/cm^2^), chicken exposed to pulsed UV light at low intensity at fluence 1.25 J/cm^2^ and chicken exposed to pulsed UV light three times at high intensity giving a fluence of 10.8 J/cm^2^. Based on a pretrial performed by the panelists, a consensus list of attributes for the profiling was developed: Smell of raw chicken (sour odor, sunburnt odor, burnt odor, metallic odor, sulfur odor, off‐odor, cloying odor, and rancid odor) and odor/taste/flavor of cooked chicken (sunburnt odor, burnt odor, sour flavor, burned flavor, metallic flavor, off‐flavor, cloying flavor, and rancid flavor). Both raw and cooked chicken fillet samples were evaluated. For the raw samples, the panelists were given 1/6 raw chicken fillet served at room temperature on white plastic cups coded by random three‐digit numbers. The cooked samples were heated (100°C, 100% steam, 30 min) in an Electrolux Air‐o‐steam oven (Combi LW 6 GN 1/1 Gas) to a core temperature of 78°C ± 3°C. After heating, the samples rested for 5 min before each panelist were served one‐fourth cooked chicken fillet in a white porcelain bowl with lid marked with a random three‐digit number, that had been preheated at 65°C. Samples were kept at 65°C for the evaluation. The panelists had unsalted crackers and lukewarm water for rinsing the palate between samples. The coded samples were evaluated in duplicate and served randomized according to sample, panelist, and replicate. Each panelist recorded their results at individual speed using an unstructured line scale with labeled endpoints ranging from no intensity (1), to high intensity (9), using the EyeQuestion Software (Logic8 BV, Elst, The Netherlands) for direct recording of data.

Changes in the quality or sensory properties of raw chicken as a result of UV light exposure were also assessed by a smaller consumer test. Twenty randomly chosen test persons were asked if they would want to use the chicken samples for dinner. In addition, they assessed the quality of the chicken on a scale ranging from very bad (1), to very good (9).

### 2.7 Dynamic headspace gas chromatography mass spectrometry

The same set of raw chicken samples used in the pretrail sensory evaluation was subjected to dynamic headspace gas chromatography mass spectrometry (GC/MS) analysis. Based on variation found both in the sensory results and the GC/MS results of the pretrial, chicken samples that showed the greatest variation were further selected for analysis of volatile organic compounds. These included: untreated control, chicken exposed to UV‐C light at fluence 0.60 J/cm^2^ (60 s at 10 mW/cm^2^) and pulsed UV light three times at high intensity giving a fluence of 10.8 J/cm^2^ treated in air, and pulsed UV light at low intensity at fluence 1.25 J/cm^2^ treated under modified atmosphere. A gas chromatography analysis was carried out on chicken samples as previously described (Olsen, Vogt, Veberg, Ekeberg, & Nilsson, 2005). Fifteen gram aliquots of homogenized sample (the samples were analyzed in duplicate) were distributed evenly in 250 ml Erlenmeyer flasks. The samples were heated to 70°C in a water bath and purged with 100 ml/min nitrogen through a Drechsel‐head for 30 min. Volatile compounds were adsorbed on Tenax GR (mesh size 60/80). Water was removed from the tubes by nitrogen flushing (50 ml/min) for 5 min in the opposite direction of sampling. Trapped compounds were desorbed at 250°C for 5 min in a Perkin Elmer Automatic Thermal Desorption System ATD400 and transferred to an Agilent 6890 GC System with an Agilent 5973 Mass selective detector, which is a quadrupole, operated in electron impact (EI) mode at 70 eV. The scan range was from 33 to 300 amu. The compounds were separated on a DB‐WAXetr column from J&W Scientific/Agilent (0.25 mm i.d., 0.5 lm film, 30 m). Helium (99.9999%) was used as carrier gas. The temperature program started at 30°C for 10 min, increased 1°C/min to 40°C, 3°C/min to 70°C, 6.5°C/min to 160°C, and 20°C/min to 230°C with a final hold time of 4 min. Integration of peaks and tentative identification of compounds were performed with HP Chemstation (G1701CA version C.00.00, Agilent Technologies, Santa Clara, CA, USA), Wiley 130 KMass Spectral and NIST98 Mass Spectral. Comparison of retention times and mass spectra of the sample peaks with those of pure standards confirmed identities of several of the components. Heptanoic acid ethyl ester was used as internal standard. System performance was checked with blanks and standard samples before, during and after the sample series, and the selected major compounds (80–100%) on a peak area basis were included in the data analysis.

### 2.8 Statistical analysis

Bacterial reductions log cfu/cm^2^ between control and UV light treated samples were calculated. Analysis of variance (ANOVA) and Tukey's multiple comparison test were used to determine statistically significant effects on the reduction by the treatments (R 3.3.2; R Core Team [2016]) using a significance level of .05. For sensory evaluation, the same analyses were performed on the descriptive sensory data from the trained panel to identify sensory attributes that discriminated between samples.

### 2.9 Weibull models

For each species, a two‐parameter Weibull distribution was fitted to the observed log reductions to produce predictive models of the effects of UV exposure. The chosen Weibull model is defined as:

$$log_{10}\left(\frac{N}{N_{0}}\right)=\frac{-1}{log_{e}(10)}{\left(\frac{f}{\alpha }\right)}^{\beta },$$

where *N*_0_ and *N* denote the number of bacteria per square cm before and after UV exposure, respectively, *f* is the UV dose (fluence), α is the scale parameter (describes how sharply the curve drops in the beginning), and β is the shape parameter (describes the shape of the curve). Common models were produced based on log reduction data for all the bacterial species.

## 3 RESULTS

### 3.1 Bacterial reductions on skinless chicken fillets

**Figure 1:**
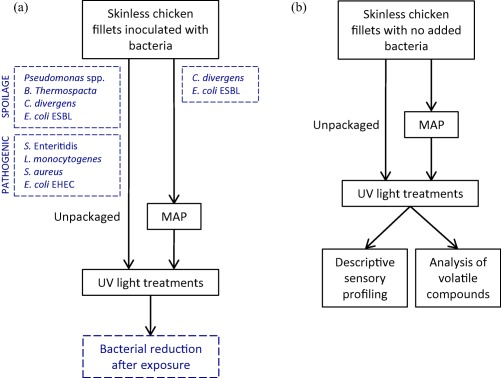
Flowchart illustrating the experimental set‐up. Reduction of bacteria on skinless chicken fillets using UV light treatments (a), and sensory analyses of chicken fillets treated with UV light (b). Chicken fillets inoculated with pathogens and bacteria often found as natural contaminants on fresh chicken meat were exposed to different UV light treatments in air, representing unpackaged chicken, and for two selected species on modified atmosphere packaged (MAP)‐chicken. The bacterial species are listed in Table 1. Sensory analyses of chicken fillets with no added bacteria were conducted after UV light treatments of both unpackaged chicken and MAP‐chicken

**Figure 2:**
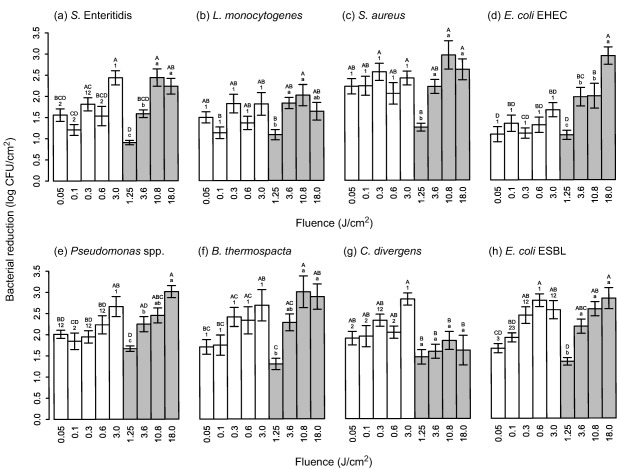
Reductions of (a) *S*. Enteritidis, (b) *L. monocytogenes*, (c) *S. aureus*, (d) enterohemorrhagic *E. coli* (EHEC), (e) *Pseudomonas* spp., (f) *B. thermospacta*, (g) *C. divergens*, and (h) ESBL‐producing *E. coli* on chicken fillet meat after continuous UV‐C (white bars) and pulsed UV light (grey bars) exposures at different fluences (J/cm^2^). The chicken samples were treated in air, representing unpackaged chicken. Three separate ANOVA were performed for each species, represented by upper case letters (comparing UV‐C and pulsed UV light treatments), numbers (comparing UV‐C light treatments) and lower case letters (comparing pulsed UV light treatments). Samples containing the same letter/number were not considered different

**Figure 3:**
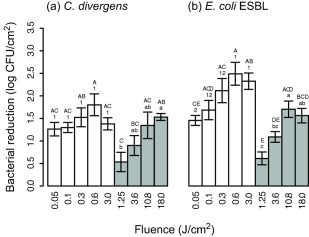
Reductions of (a) *C. divergens* and (b) ESBL‐producing *E. coli* on MAP‐chicken exposed to continuous UV‐C (white bars) and pulsed UV light (grey bars) at different fluences (J/cm^2^). A gas mixture of 60% CO_2_ and 40% N_2_ and a UV permeable top film was used for the packages. Three separate ANOVA were performed for each species, represented by upper case letters (comparing UV‐C and pulsed UV light treatments), numbers (comparing UV‐C light treatments) and lower case letters (comparing pulsed UV light treatments). Samples containing the same letter/number were not considered different

We investigated the effect of UV‐C and pulsed UV light against microbial flora associated with fresh, skinless chicken fillets. An overview of the experimental set‐up is shown in Figure 1. Resulting bacterial log reductions cfu/cm^2^ of the food pathogens *S*. Enteritidis, *L. monocytogenes*, *S. aureus* and EHEC, and chicken spoilage bacteria *Pseudomonas* spp., *B. thermospacta*, *C. divergens*, and ESBL‐producing *E. coli* applied to chicken meat surface are shown in Figures 2 and 3 and Supporting Information Table S1.

UV‐C light exposure with fluences from 0.05 to 3.0 J/cm^2^ (10 mW/cm^2^, from 5 to 300 s) in air, gave the largest reduction of 2.8 log for *C. divergens* after the highest fluence treatment, while only 1.7 log reduction was obtained for EHEC. The lowest fluence level gave up to 2.2 log reduction for *S. aureus*, and EHEC was reduced the least with 1.1 log. By comparing UV‐C light results using ANOVA within each species, some of the shorter treatments were considered statistically different from the treatments of longer duration for *S*. Enteritidis (Figure 2a, range 1.6–2.4 log), *Pseudomonas* spp. (2e, 2.0–2.7 log), *C. divergens* (2g, 1.9–2.8 log), and ESBL‐producing *E. coli* (2h, 1.7–2.8 log), while none of the treatments were statistically different from each other for *L. monocytogenes* (2b, 1.5–1.8 log), *S. aureus* (2c, 2.2–2.6 log), EHEC (2d, 1.1–1.7 log), and *B. thermospacta* (2f, 1.7–2.7 log).

Sensitivities against pulsed UV light, where fluences from 1.25 to 18.0 J/cm^2^ were used, seemed to be more similar between the different species than for UV‐C light. Reductions after pulsed UV light exposure in air at the highest fluences (10.8 and 18.0 J/cm^2^) ranged from 1.6 log for *L. monocytogenes* and *C. divergens* to 3.0 log for *S. aureus, Pseudomonas* spp. and *B. thermospacta*. For the low fluence exposure of 1.25 J/cm^2^, reductions ranged from 0.9 log for *S*. Enteritidis to 1.7 log for *Pseudomonas* spp. ANOVA on the pulsed UV light results within each species defined the treatment at low fluence statistically different from some or all of the higher intensity treatments, thus increased reduction was obtained by increasing the UV dose. The range of reduction was 0.9–2.4 log for *S*. Enteritidis (Figure 2a), 1.1–2.0 log for *L. monocytogenes* (2b), 1.3–3.0 log for *S. aureus* (2c), 1.1–2.9 log for EHEC (2d), 1.7–3.0 log for *Pseudomonas* spp. (2e), 1.3–3.0 log for *B. thermospacta* (2f), and 1.3–2.8 log for ESBL‐producing *E. coli* (2h). *C. divergens* deviated from this pattern, for which none of the treatments were considered statistically different from each other and reductions ranged from 1.5 to 1.8 log (Figure 2g).

In the *in vitro* illumination experiments of petri dishes, the UV light treatments inactivated all the bacterial species by 5–7 log, except from *L. monocytogenes* that was able to withstand the low fluence 1.25 J/cm^2^ treatment with pulsed UV light better than the other species, showing approximately 4 log reduction (not shown).

Bacterial reductions after exposure with UV‐C and pulsed UV light against *C. divergens* and ESBL‐producing *E. coli* on MAP‐chicken, are shown in Figure 3 and Supporting Information Table S1. Samples were stored under an anaerobic atmosphere with 60% CO_2_ and 40% N_2_, and the UV permeable top film allowed for UV light exposure of intact packages. *C. divergens* reduction after UV‐C light treatments ranged from 1.3 to 1.8 log, and after pulsed UV light treatments from 0.5 to 1.5 log. The UV‐C light treatments at the lowest fluences (0.05, 0.1, 0.3 J/cm^2^) resulted in approximately 0.7 log lower reduction on MAP‐chicken compared with unpackaged chicken, and 1.4 log lower reduction was seen for the highest fluence treatment (3.0 J/cm^2^). ANOVA on the UV‐C light results confirmed the observed differences statistically (results not shown). After pulsed UV light exposure, reductions were similar for MAP‐chicken and unpackaged chicken samples for the highest fluences (10.8 and 18.0 J/cm^2^), while for fluences of 1.25 and 3.6 J/cm^2^, 0.9 and 0.7 log lower reductions, respectively, were seen on MAP‐chicken, which were confirmed statistically by ANOVA (not shown). Reduction of ESBL‐producing *E. coli* after UV‐C light treatments ranged from 1.5 to 2.5 log, and after pulsed UV light treatments from 0.6 to 1.7 log. ANOVA on the UV‐C light results confirmed statistically that reductions on MAP‐chicken and unpackaged chicken samples were similar (not shown). For pulsed UV light, lower reductions were seen for the MAP‐chicken samples regardless of UV dose, 0.7, 1.1, 0.9, and 1.3 log lower reductions for fluences of 1.25, 3.6, 10.8, and 18.0 J/cm^2^, respectively, confirmed statistically by ANOVA (not shown).

The applied UV light up to 10.8 J/cm^2^ did not impair the oxygen barrier properties and structural integrity of the UV permeable top film, and the O_2_ concentrations of the trays increased from approximately 0.12 ± 0.03% at packaging to 0.69 ± 0.02% after 21 days, and were similar for the different UV light treatments and the untreated control. Scanning electron microscopy analysis showed no structural damages to the UV treated films (not shown). The ability of the film to transmit UV light was measured as 80.5% at 254 nm, which was compensated for by increasing the UV doses accordingly in the illumination experiments.

### 3.2 Weibull models describing bacterial reduction

**Figure 4:**
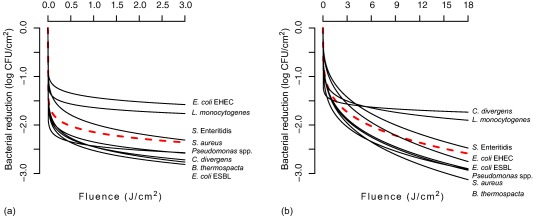
Weibull models for bacterial log reduction as a function of UV exposure. Models for each species (black continuous line) and common models (red dotted line) are shown for bacterial reduction on unpackaged chicken fillet meat after (a) continuous UV‐C and (b) pulsed UV light exposures at different fluences (J/cm^2^)

Weibull models created to predict the log reduction patterns for the different bacterial species are shown in Figure 4 and parameters for the models are listed in Table 2. *RMSE* values indicating the goodness of fit, were the lowest for *S. aureus* exposed to UV‐C light (0.20) and the highest for *Pseudomonas* spp. exposed to pulsed UV light (0.55). Determination coefficient (*R* ^2^) values ranged from 0.41 to 0.80 for UV‐C light and from 0.47 to 0.89 for pulsed UV light. Since *R* ^2^ indicates the proportion of variation in log reduction explained by the fitted Weibull model, a value approaching 1 would signify perfect predictability. Since all of the ß (shape parameter) values were less than 1, the Weibull fits of the reduction data were concave upward. The highest ß values were obtained for EHEC and *S*. Enteritidis (0.32 and 0.31, respectively) for pulsed UV light. The α (scale parameter) values were very small, implying concentrated distribution, as seen by how sharp the curve drops in the beginning. There was a noticeable difference between the two UV methods, where higher α values were obtained for UV‐C light than for pulsed UV light, with *C. divergens* as an exception. Common models based on log reduction values for all the species gave a good fit for the majority of the species, but for *L. monocytogenes* exposed to both UV‐C and pulsed UV light, reduction was overestimated. The same was seen for EHEC exposed to UV‐C light and *C. divergens* exposed to pulsed UV light.

**Table 2:** Parameters for Weibull models predicting bacterial reduction on chicken fillet meat after continuous UV‐C and pulsed UV light exposures, and goodness‐of‐fit parameters of the models

	Bacterial species	α	β	*RMSE*	*R* ^2^
Continuous UV‐C light	*E. coli* EHEC	2.03E‐06	0.09	0.31	.75
	*L. monocytogenes*	2.02E‐09	0.07	0.47	.41
	*S*. Enteritidis	2.35E‐05	0.14	0.41	.64
	*S. aureus*	2.22E‐15	0.05	0.20	.76
	*Pseudomonas* spp.	2.86E‐09	0.09	0.39	.68
	*C. divergens*	1.45E‐08	0.10	0.37	.74
	*B. thermospacta*	1.66E‐07	0.11	0.31	.80
	*E. coli* ESBL	1.65E‐08	0.10	0.38	.74
	All	9.89E‐09	0.09	0.53	.25
Pulsed UV light	*C. divergens*	3.79E‐10	0.06	0.29	.86
	*L. monocytogenes*	2.27E‐04	0.13	0.37	.63
	*S*. Enteritidis	6.32E‐02	0.31	0.42	.79
	*E. coli* EHEC	5.29E‐02	0.32	0.41	.79
	*E. coli* ESBL	7.58E‐03	0.24	0.28	.89
	*Pseudomonas* spp.	1.31E‐03	0.20	0.55	.71
	*S. aureus*	6.61E‐03	0.24	0.47	.47
	*B. thermospacta*	9.21E‐03	0.26	0.37	.82
	All	6.23E‐03	0.22	0.54	.46

### 3.3 Sensory evaluation of UV light treated chicken

**Figure 5:**
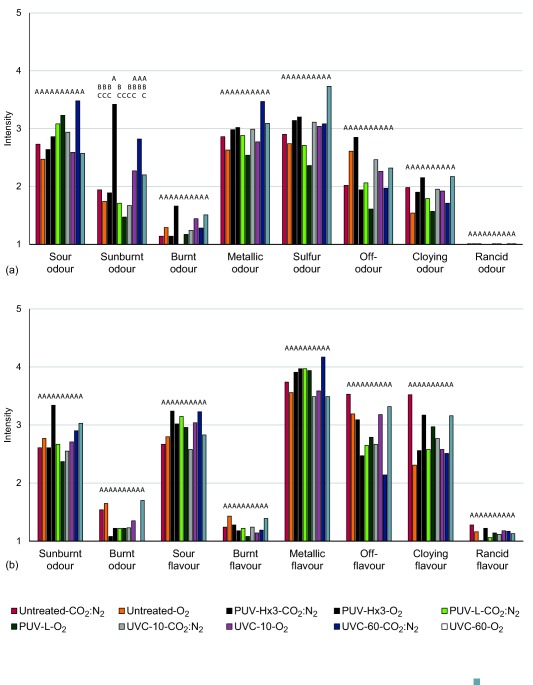
Sensory analysis of (a) raw chicken fillet samples and (b) cooked chicken fillet samples. Chicken samples were exposed to continuous UV‐C light at 10 mW/cm^2^ for 10 s (UVC‐10) and 60 s (UVC‐60), giving fluences of 0.1 J/cm^2^ and 0.60 J/cm^2^, respectively, and pulsed UV light to a low pulse with fluence of 1.25 J/cm^2^ (PUV‐L) and three times to a high pulse giving a fluence of 10.8 J/cm^2^ (PUV‐Hx3), both in air (O_2_) and anaerobic (CO_2_ : N_2_) atmospheres, representing unpackaged chicken and MAP‐chicken, respectively. The intensities of different odors of raw samples and odor/taste/flavor of cooked samples were registered, 1 = no intensity and 9 = high intensity. The letters above the columns indicate grouping according to ANOVA and Tukey multiple comparison test. Samples with the same letter are considered being equal for the specific property

Changes in quality or sensory properties of chicken fillets as a result of UV light treatments were assessed by 10 trained assessors. Their evaluation results are shown in Figure 5, where raw chicken samples were evaluated for odor and cooked chicken samples for odor/taste/flavor. A statistically significant difference between the samples was only registered for the odor characterized as sunburnt (*p* < .001), which is associated with that of sunburnt human skin. Most notably, treatment with the highest dose of pulsed UV light (10.8 J/cm^2^) in air gave the highest intensity of the sunburnt odor (sensory intensity value score of 3.4). After cooking, this effect of the UV light treatment could not be detected. From the consumer test, UV light exposed raw chicken fillet samples assessed by 20 random consumers could not be differentiated from untreated control samples (data not shown). By visual inspection, the color stability was not affected by the treatments at the doses used (data not shown).

Denaturation of proteins in chicken has been considered to be initiated at temperatures higher than 56°C (Murphy, Marks, & Marcy, 1998). Only minor elevation of the temperature was observed, 2.5–4.0°C and 4.0–6.5°C for UV‐C light treatments at fluences 0.6 J/cm^2^ and 3.0 J/cm^2^, respectively, and 0.5–2.5°C and 2.5–3.5°C for pulsed UV light treatments at fluences 10.8 and 18.0 J/cm^2^, respectively. The rise in surface temperature was only temporary since the surface was rapidly cooled by the low temperature of the interior of the chicken fillet.

### 3.4 Volatile organic compounds

**Figure 6:**
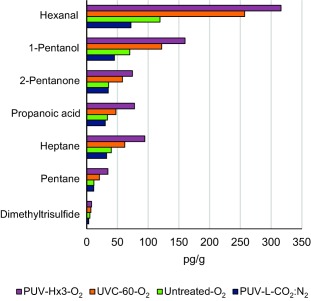
Volatile organic compounds from chicken which showed an increase in concentration (pg/g) as a result of exposure to UV light. The samples included were chicken exposed to pulsed UV light at low intensity at fluence 1.25 J/cm^2^ (PUV‐L) treated under anaerobic (CO_2_:N_2_) atmosphere (MAP‐chicken), an untreated control (Untreated), chicken exposed to UV‐C light at 10 mW/cm^2^ for 60 s (UVC‐60) giving a fluence of 0.60 J/cm^2^ and pulsed UV light three times at high intensity (PUV‐Hx3) giving a fluence of 10.8 J/cm^2^ treated in air (O_2_). The precision of replicate measurements were within 15%

Nearly 100 different volatile organic compounds were detected by dynamic headspace/GC‐MS in the raw chicken samples that were analyzed, of which approximately 70 compounds could be identified. The major compounds were ketones (C2–C5, C7), alcohols (C2–C8), acids (C2–C7), fatty and nonfatty aldehydes (C2–C9), hydrocarbons (C5–C7), and sulfides. Only a few compounds were observed to increase in concentration as a result of exposure to UV light. This included dimethyltrisulfide, pentane, heptane, propanoic acid, 2‐pentanone, 1‐pentanol, and hexanal (Figure 6). Linear correlation with the odor scores were calculated, and gave correlations with the sunburnt odor scores as follows: dimethyltrisulfide *r* = .70 (*p* < .01), 2‐pentanone *r* = .95 (*p* < .0025), 1‐pentanol *r* = .91 (*p* < .005), pentane (*r* = .92, *p* < .005), heptane (*r* = .81, *p* < .01), propanoic acid (*r* = .98, *p* < .001), and hexanal (*r* = .81, *p* < .01). The sample in which all the compounds increased the most, was chicken exposed to pulsed UV light at fluence 10.8 J/cm^2^ treated in air.

## 4 DISCUSSION

### 4.1 Effect of UV treatment on inoculated bacteria

There are large differences between the conventional continuous UV‐C light and pulsed UV light with respect to wavelengths, intensities, and exposure times. In this work, we have compared the efficacy of continuous UV‐C light and pulsed UV light in reducing bacteria on chicken fillet. We used multi strain mixtures of the same species and bacterial cells that were in the same state during the different treatments. In earlier studies, single strains were often used which may not show reductions representative for the species. Differences in reduction within species have been reported, and state of the cells can influence the sensitivity to UV light (Farrell et al., 2010; Haughton et al., 2011b). To avoid possible changes in sensory perception, it is desirable to maximize bacterial reduction without treating the surface of a product more than necessary. Treatment levels employed for both UV methods were practical and relevant within industrial production, from weak exposures resulting in limited bacterial reduction, up to levels exceeding the maximum permitted dose by the FDA for pulsed UV light (FDA, 2010). The fluences are not directly comparable between the two methods, since the different wavelengths in the UV spectrum have different germicidal effectiveness (Bintsis, Litopoulou‐Tzanetaki, & Robinson, 2000). For UV‐C exposure at 0.05 J/cm^2^, the germicidal effect was comparable to a fluence of 1.25 J/cm^2^ for the pulsed UV light. UV‐C light showed a higher germicidal effect when the same fluence was employed for the two methods, which can be explained by most of the energy being emitted at 254 nm, where the germicidal effect is close to the maximum (Bintsis et al., 2000).

In the range tested, a limited dose‐response effect was observed, likely caused by shading effects of the irregular surface structure of the chicken fillet. The increase in reduction with increasing dose was though more apparent for the pulsed UV light. Any substance between the light source and the bacterium that absorbs light will impair the decontamination process (Gomez‐Lopez et al., 2007). Even when a surface appears smooth to the naked eye, it may harbor crevices and cracks where bacteria are shielded against direct exposure, and bacteria may also be covered by protein or other organic matrices. Moreover, the average size of a bacterium is approximately 1 µm × 2 µm, and although its spreading was carried out carefully, it is practically impossible to avoid some overlapping. A shielding effect for colonies of *L. monocytogenes* growing on petri dishes where the upper cells of a colony appeared to protect the lower cells has previously been described (Gomez‐Lopez et al., 2005). At high fluence rates, the light should be able to penetrate deeper, but still, the efficiency of using UV light for decontamination of foods is lower than when tested on smooth surfaces. Reductions of 5–7 log achieved on agar in petri dishes was in accordance with previous reports (Farrell et al., 2010; Gomez‐Lopez et al., 2005; Paskeviciute et al., 2011; Rowan et al., 1999), and the observed higher resistance of *L. monocytogenes* to pulsed UV light, reduced only 4 log after treatment at low fluence of 1.25 J/cm^2^, has also been reported previously (Gomez‐Lopez et al., 2005; Lasagabaster & de Maranon, 2012). In general, the reductions of inoculated bacteria on chicken fillet surface observed in this study correlated well with previous findings, both for UV‐C (Chun et al., 2010; Haughton et al., 2011a; Isohanni & Lyhs, 2009; Sommers et al., 2016) and for pulsed UV light (Keklik et al., 2010; Paskeviciute et al., 2011), including for *C. divergens*, *Pseudomonas* spp., and *B. thermospacta*, for which previous reports on UV light inactivation on food surfaces does not exist or are scarce. EHEC seemed to resist the UV‐C light treatments better than ESBL‐producing *E. coli*, and better than the other species tested as well.

The Weibull distribution is suitable for the analysis of bacterial reduction (Chen, 2007; Keklik, Demirci, Puri, & Heinemann, 2012; Martin et al., 2007; Ugarte‐Romero, Feng, Martin, Cadwallader, & Robinson, 2006; van Boekel, 2002), and was previously demonstrated to be more successful than models such as the log‐linear model and first‐order kinetic model (Chen, 2007; Keklik, Demirci, et al., 2012; Martin et al., 2007). The model seemed to be a useful tool to describe the reduction patterns and give clues to how pathogens and spoilage bacteria on chicken fillet surfaces are likely to respond to UV light treatments. The Weibull fits of the reduction data were concave upward, indicating that exposed cells were destroyed and that the more resistant cells or those shaded from exposure were left undamaged.

To our knowledge, studies on UV light treatment of intact packages of MAP‐chicken fillet for reducing bacteria on the chicken surface have previously not been reported. UV light reduction of bacteria on various packaging materials have, however, been studied (Haughton et al., 2011b), and vacuum‐packaged chicken breast inoculated with *Salmonella* Typhimurium treated with pulsed UV light were shown to give about 2 log reduction, but with double the exposure time (30 s) in comparison with unpackaged samples (15 s) (Keklik et al., 2010). The additional bacterial reduction obtained on ready packaged chicken fillet product would increase shelf life and safety. Treatment after packaging should be simple to implement at industrial packaging lines without reductions in production efficiency.

### 4.2 Sensory quality of the chicken fillets

Meat exposed to UV light can develop off‐flavors caused by the absorption of ozone and oxides of nitrogen, or because of photochemical effects on the lipid fractions of the meat (Bintsis et al., 2000). Lipid oxidative rancidity is regarded as the most important nonmicrobial factor responsible for meat deterioration, resulting in adverse changes in appearance, texture, odor, and flavor (Frankel, 1998). An increase in fatty aldehydes due to lipid oxidation during irradiation of poultry meat has been documented (Du, Ahn, Nam, & Sell, 2000, 2001; Du, Hur, Nam, Ismail, & Ahn, 2001; Kim, Nam, & Ahn, 2002). The major fatty aldehyde hexanal is a typical volatile secondary lipid oxidation product (Beltran, Pla, Yuste, & Mor‐Mur, 2003; Jayasena, Ahn, Nam, & Jo, 2013; Shi & Ho, 1994). Although we observed an increase in the concentration of hexanal, particularly for unpackaged chicken exposed to UV light, no significant effect was found on the corresponding rancid‐related sensory attributes in the professional sensory evaluation. This suggests that lipid oxidation does not have a negative impact on the perceived odor and flavor of the chicken meat at the applied UV doses. The higher intensity of the sunburnt odor for chicken exposed to the most intense dose of pulsed UV light, does, however, seem to pose restrictions on the upper limit of treatment of unpackaged chicken. The sensory intensity value was though only 3.4, which is considered relatively low, and for lower doses relevant in industrial application, the odor should not be a problem. Detected changes in concentrations of volatile compounds correlated well with the sensory observations. Increased levels were seen in unpackaged chicken after UV light exposure. Hydrocarbons may be generated during irradiation of poultry meat (Du, Ahn, et al., 2000, 2001; Du, Hur, et al., 2001; Kim et al., 2002), where increased concentrations of propanol and butanol have been documented (Du et al., 2000, 2001; Du, Hur, et al., 2001). In accordance, we detected increased levels of pentane, heptane and 1‐pentanol. Sulfur compounds with low odor thresholds are important to odor associated with irradiation (Angelini, Merritt, Mendelsohn, & King, 1975; Batzer & Doty, 1955; Patterson & Stevenson, 1995). Dimethyltrisulfide, although only detected in small amounts in unpackaged chicken after UV light exposure, was reported by Patterson and Stevenson (Patterson & Stevenson, 1995) to be the most potent off‐odor compound in irradiated raw chicken. Other compounds that showed an increase and which character could be associated with sunburnt/irradiated odor and flavor, were 2‐pentanone (roasted sweet), and 1‐pentanol (roasted meat) (Brewer, 2009). Together these three compounds likely contribute to the sensory perceived sunburnt odor. Irradiation of poultry meat is though based on irradiation by electrons using an accelerator, representing far higher dose in terms of energy exposure per area compared to our applied UV doses, thus the results may not be directly comparable. Paskeviciute et al. (2011) investigated chemical changes in pulsed UV light treated chicken breasts, and reported that the intensity of lipid peroxidation in control and treated chicken samples differed in 0.16 mg malondialdehyde per kilogram of chicken meat. However, taste panelists did not observe any changes in organoleptic properties of treated raw chicken, chicken broth or cooked chicken meat in comparison with control. Although treated raw chicken samples could not be differentiated from an untreated control sample by the 20 random chosen consumers in the present study, more extensive consumer studies could aid in determining whether such UV light treatments are acceptable.

The color of raw or cooked poultry meat is by origin pale with a low content of the muscle pigment myoglobin. Furthermore, the color of raw meat is dependent on the oxidation state of myoglobin (Mugler & Cunningham, 1972; United States Department of Agriculture, 2013). Chicken breasts exposed to high doses of UV light was previously reported to turn darker, show more redness and a slight increasing amount of yellow coloration (Park & Ha, 2015). The color of the chicken fillets was not affected by the treatments at the doses used in our experiments, as in agreement with other reports (Chun et al., 2010; Haughton et al., 2011a). Together these results indicate that sensory and quality changes are small or negligible both after UV‐C and pulsed UV light treatments.

### 4.3 Advantages and disadvantages of continuous UV‐C and pulsed UV treatments

Both UV‐C and pulsed UV light treatments provide effective tools for reduction of microorganisms. They are rapid and efficient nonchemical, nonionizing, and nonthermal surface decontamination treatments and can be used in continuous processing. The methods have been shown as effective technologies for decontamination of stainless steel conveyors and surfaces in the production environment (Haughton et al., 2011b; Sommers, Sites, & Musgrove, 2010). They can be used on foods and synergistically with other treatments (Mukhopadhyay & Ramaswamy, 2012). The methods require little energy use, are easy to implement and require no increase in work load. UV light is safe to apply, but some precautions have to be taken to avoid exposure of workers to light and to evacuate any ozone generated by the shorter UV wavelengths (Gomez‐Lopez et al., 2007). The effect of both UV‐C and pulsed UV light is impaired in opaque matter, where bacteria are shielded from direct exposure such as by food surface topography, organic matter, or by other bacteria. The UV light treatments of this study did not alter the properties of the EVOH film used, as was also the case with polyethylene, polypropylene and polyvinyldichloride films (Tarek, Rasco, & Sablani, 2015). The top film used transmitted approximately 80% of the UV light, while in previous studies, films with polypropylene and polyethylene barrier layers transmitted 75% (Keklik, Demirci, & Puri, 2009) and 72% (Keklik et al., 2010), respectively, of pulsed UV light at 1.27 J/cm^2^. By using a packaging film with a high UV transmission, the chicken fillets could be packaged before the UV light treatment, thereby avoiding the risk of recontamination. Both methods would be beneficial for large scale industrial UV decontamination operations. UV‐C light treatment is a low cost strategy with low maintenance (Keklik, Krishnamurthy, & Demirci, 2012). The treatment time is somewhat longer in comparison with pulsed UV light treatment, and therefore the equipment may require more space if installed over for example a conveyor belt. Pulsed UV light provides rapid decontamination, but involves equipment that is more elaborate. The xenon flash lamps used for pulsed UV light are also more environment friendly than the mercury‐vapor lamps typically used in UV‐C light treatment (Gomez‐Lopez et al., 2007).

## 5 CONCLUSION

Despite good hygiene practices during production of fresh meat, contamination of carcasses with pathogens and spoilage bacteria cannot be completely prevented. There is pressure on the food industry for nutritious, fresh and healthy food products, to maximize the shelf life of the products, and for reducing costs and waste. Antimicrobial interventions that effectively reduce the bacterial load are feasible in slaughter and product processing. They should be safe, economic, and easy to handle. Also, interventions should not change the organoleptic quality of the food and should be widely accepted by consumers. The exposure of raw chicken fillet surface to various doses of UV‐C or pulsed UV light proposed in this work represents useful alternatives for reducing the viability of pathogenic and spoilage bacteria on this product.

## CONFLICT OF INTEREST

The authors declare that there is no conflict of interest regarding publication of this paper.

## Supporting information

Additional Supporting Information may be found online in the supporting information tab for this article.

Supporting Information
