# Supplementary material for: Chicken fillets subjected to UV‐C and pulsed UV light: Reduction of pathogenic and spoilage bacteria, and changes in sensory quality
Source: J Food Saf. 2017 Nov 23;38(1):e12421. doi: 10.1111/jfs.12421 (PMC6084340; doi:10.1111/jfs.12421)
Supplement: Supplementary file 1 — Supporting Information [file JFS-38-na-s001.pdf]

# SUPPORTING INFORMATION

**TABLE S1** Bacterial reductions log CFU/cm$^2$ on chicken fillet meat after continuous UV-C and pulsed UV light treatments at different fluences (J/cm$^2$)

| | Continuous UV-C light treatment[a], J/cm$^2$ | | | | | Pulsed UV light treatment[a], J/cm$^2$ | | | |
|---|---|---|---|---|---|---|---|---|---|
| **Bacterial species** | **0.05** | **0.1** | **0.3** | **0.6** | **3.0** | **1.25** | **3.6** | **10.8** | **18.0** |
| ***S.* Enteritidis** | 1.56 (0.15) | 1.21 (0.13) | 1.81 (0.16) | 1.53 (0.23) | 2.44 (0.17) | 0.90 (0.05) | 1.58 (0.09) | 2.44 (0.20) | 2.23 (0.19) |
| ***L. monocytogenes*** | 1.49 (0.13) | 1.13 (0.14) | 1.82 (0.22) | 1.36 (0.16) | 1.81 (0.27) | 1.08 (0.12) | 1.83 (0.13) | 2.01 (0.25) | 1.63 (0.21) |
| ***S. aureus*** | 2.22 (0.18) | 2.23 (0.22) | 2.56 (0.20) | 2.05 (0.26) | 2.41 (0.16) | 1.25 (0.09) | 2.21 (0.16) | 2.95 (0.33) | 2.61 (0.25) |
| ***E. coli* EHEC** | 1.08 (0.18) | 1.34 (0.19) | 1.10 (0.12) | 1.30 (0.18) | 1.65 (0.17) | 1.06 (0.11) | 1.96 (0.22) | 1.98 (0.29) | 2.93 (0.21) |
| ***Pseudomonas* spp.** | 2.01 (0.10) | 1.85 (0.20) | 1.95 (0.15) | 2.23 (0.21) | 2.66 (0.24) | 1.67 (0.06) | 2.25 (0.18) | 2.45 (0.18) | 3.01 (0.14) |
| ***B. thermospacta*** | 1.70 (0.17) | 1.74 (0.24) | 2.41 (0.22) | 2.33 (0.32) | 2.68 (0.37) | 1.30 (0.13) | 2.28 (0.20) | 3.00 (0.37) | 2.88 (0.30) |
| ***C. divergens*** | 1.90 (0.16) | 1.95 (0.25) | 2.32 (0.14) | 2.04 (0.14) | 2.82 (0.14) | 1.46 (0.17) | 1.59 (0.17) | 1.84 (0.21) | 1.61 (0.35) |
| ***E. coli* ESBL** | 1.65 (0.11) | 1.91 (0.11) | 2.43 (0.20) | 2.79 (0.15) | 2.56 (0.22) | 1.34 (0.09) | 2.17 (0.17) | 2.58 (0.17) | 2.83 (0.25) |
| ***C. divergens* MAP[b]** | 1.26 (0.15) | 1.30 (0.11) | 1.52 (0.21) | 1.80 (0.24) | 1.38 (0.14) | 0.54 (0.21) | 0.90 (0.22) | 1.35 (0.29) | 1.53 (0.08) |
| ***E. coli* ESBL MAP[b]** | 1.46 (0.11) | 1.69 (0.22) | 2.12 (0.27) | 2.49 (0.25) | 2.33 (0.18) | 0.61 (0.14) | 1.09 (0.12) | 1.70 (0.18) | 1.56 (0.16) |

[a]Standard error values are shown in brackets

[b]MAP refers to modified atmosphere packaging

For Review Only
